# Coexistence of Rheumatoid Arthritis, Cerebrovascular Disease, and Alzheimer’s Disease: A Case Study With Genetic Insights

**DOI:** 10.7759/cureus.104395

**Published:** 2026-02-27

**Authors:** Andrey Frolov, Maurice Maglasang, Miguel Guzman, M. Scott Echols, Daniel T Daly

**Affiliations:** 1 Department of Surgery, Center for Anatomical Science and Education, Saint Louis University School of Medicine, Saint Louis, USA; 2 Department of Osteopathic Medicine, New York Institute of Technology College of Osteopathic Medicine at Arkansas State University, Jonesboro, USA; 3 Department of Pathology, Saint Louis University School of Medicine, Saint Louis, USA; 4 Department of Veterinary Medicine, Scarlet Imaging, Murray, USA

**Keywords:** alzheimer’s disease, cerebrovasculature, dementia, rheumatoid arthritis, whole exome sequencing (wes)

## Abstract

To gain new insights into the molecular underpinnings of coexisting rheumatoid arthritis (RA), cerebrovascular disease (cVD), and Alzheimer’s disease (AD), we performed postmortem neuropathological examination and genetic screening of two individuals. The first individual (donor 1, D1) was a 74-year-old man who was diagnosed with both RA and AD and who also underwent hip replacement surgery bilaterally. The second individual (donor 2, D2) was a 90-year-old man with a reported diagnosis of RA, as well as two left hip replacements. A thorough histochemical (hematoxylin and eosin, H&E) and immunohistochemical (β-amyloid and tau protein) examination of D1 and D2 brains revealed the presence of AD-related pathology in both individuals, with AD stages being mild in D1 and intermediate in D2. The cVD-related pathology was also evident in both cases and was characterized by several microbleeds indicative of a compromised blood-brain barrier (BBB) integrity. Blood vessel wall thickening, a characteristic of arteriosclerosis, was significant in D1 but minor in D2. Therefore, the earlier RA diagnoses, along with the results of the neuropathological examination, indicated the coexistence in the donors of three major diseases: RA, cVD, and AD. The whole exome sequencing (WES) of DNA procured from D1 and D2 performed on the next-generation sequencing (NGS) Illumina platform (San Diego, CA) was followed by a very stringent bioinformatics analysis that yielded multiple genes with rare (minor allele frequency {MAF} ≤ 0.01) genetic pathological/deleterious variants associated with RA, cVD, and AD. Seven of those genes, *AQP7*, *ARSD*, *FAM160A1*, *HYDIN*, *IGSF3*, *OTOP1*, and *PRSS1*, were shared between D1 and D2, with all but *FAM160A1* having identical variants in both donors. Intriguingly, the subsequent analysis of the respective literature indicated that *FAM160A1*, *IGSF3*, and *PRSS1* were pleiotropic as they could be linked to all three coexisting diseases: RA, cVD, and AD. Altogether, the data presented herein are consistent with the notion that AD, cVD, and RA, when they coexist in humans, could be underpinned by a combination of polygenic and pleiotropic factors. Yet, a significant number of affected genes in the donors associated with bone and cartilage physiology point toward the possibility of joints being also damaged directly and independently of RA.

## Introduction

Three conditions affecting elderly individuals that are often observed concurrently are arthritis, cerebrovascular disease (cVD), and dementia [1]. Emerging evidence reveals the existence of a strong association between these three diseases. Autoimmune diseases, such as rheumatoid arthritis (RA), confer a significantly increased risk of developing Alzheimer’s disease (AD) [2,3]. Both RA and cVD were also identified as risk factors for developing AD [4], and cVD was shown to play a crucial role in dementia’s pathophysiological mechanism(s) [5]. Additionally, RA confers a significantly increased risk for cVD morbidity [6]. Though the specific mechanism linking these pathologies has yet to be uncovered, the inflammation associated with RA can impair the integrity of the blood-brain barrier (BBB), thereby advancing the pathogenesis of AD and cVD [7-9].

Over 30 genetic loci have been associated with RA and more than 70 with AD [10-12]. Yet, in cVD, conventional vascular risk factors such as hypertension and cigarette smoking account for only a minor causative effect on cVD, suggesting that genetics may play a major role in the cVD development [13]. Therefore, it would be reasonable to suggest that unrevealing further the role of genetic mutations in the pathogenesis of RA, cVD, and AD, especially in their concurrent state, could provide a better understanding of how these diseases are interconnected and governed by biological pathways including those underpinned by pleiotropic mechanism(s), whereby a single gene influences multiple phenotypic traits [14].

This article was previously presented in part as a meeting abstract at the 2023 Society for Neuroscience Annual Meeting on November 14, 2023.

## Case presentation

Body donors

Donor 1 (D1) is a 74-year-old man who was diagnosed with both AD and RA and underwent hip replacement surgery bilaterally. The cause of death (COD) in D1 was listed as AD-type dementia. Donor 2 (D2) is a 90-year-old man with a reported diagnosis of RA, as well as two left hip replacements. The COD for D2 was community-acquired pneumonia.

Neuropathological examination

**Figure 1:**
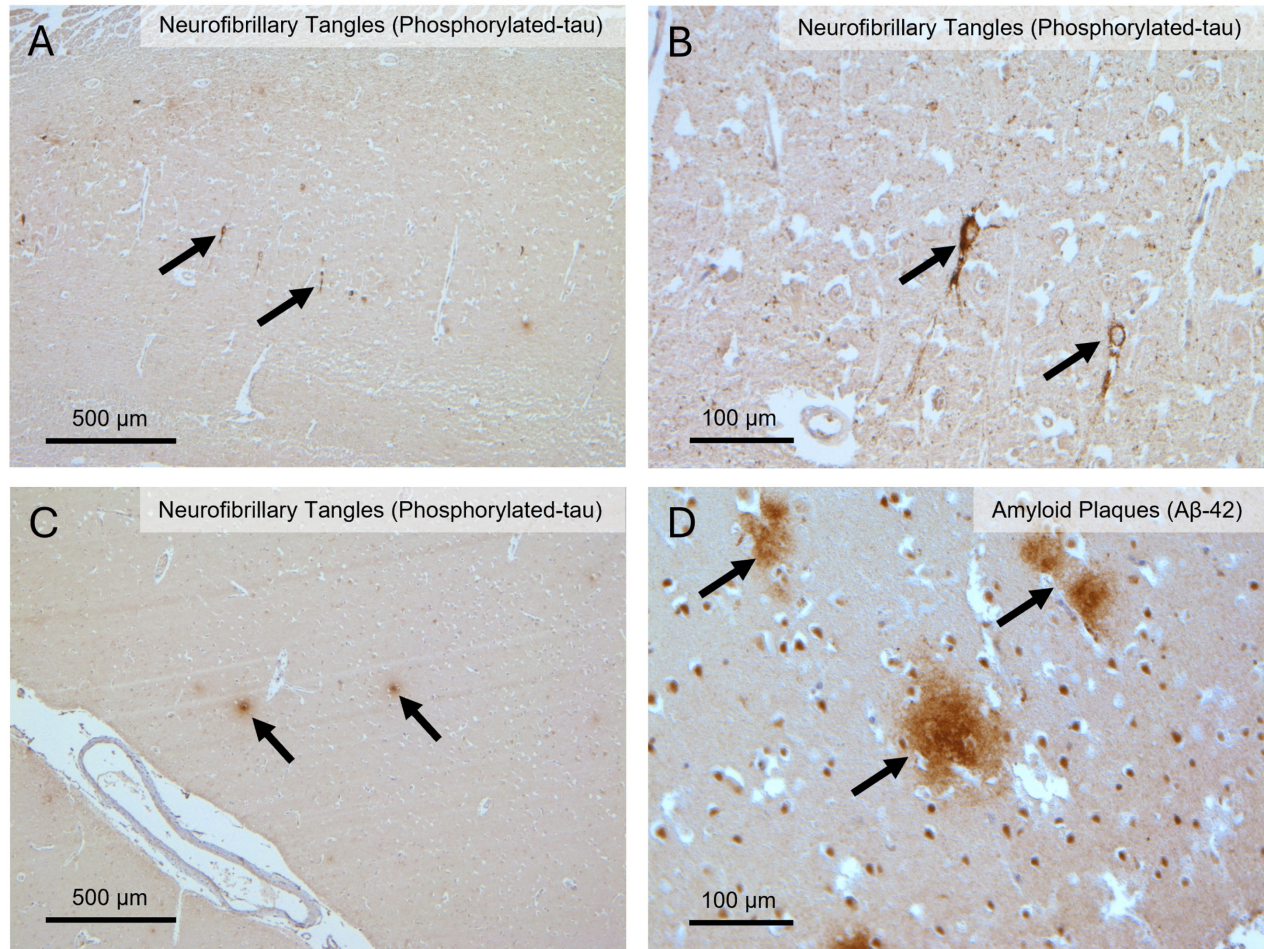
Alzheimer’s disease-related pathology in the hippocampus and cerebral hemisphere of donor 1 (D1). (A) Low-magnification hippocampal section showing scattered phosphorylated tau-positive neurofibrillary tangles within the neuropil (black arrows). (B) High-magnification hippocampal image highlighting intraneuronal phosphorylated tau-positive neurofibrillary tangles with characteristic fibrillar morphology (black arrows). (C) Low-magnification cerebral hemisphere section demonstrating focal phosphorylated tau-positive neurofibrillary tangles within cortical layers (black arrows). (D) High-magnification cerebral hemisphere section stained for Aβ-42 showing extracellular amyloid plaques with dense core morphology (black arrows). Aβ-42: amyloid beta 42

**Figure 2:**
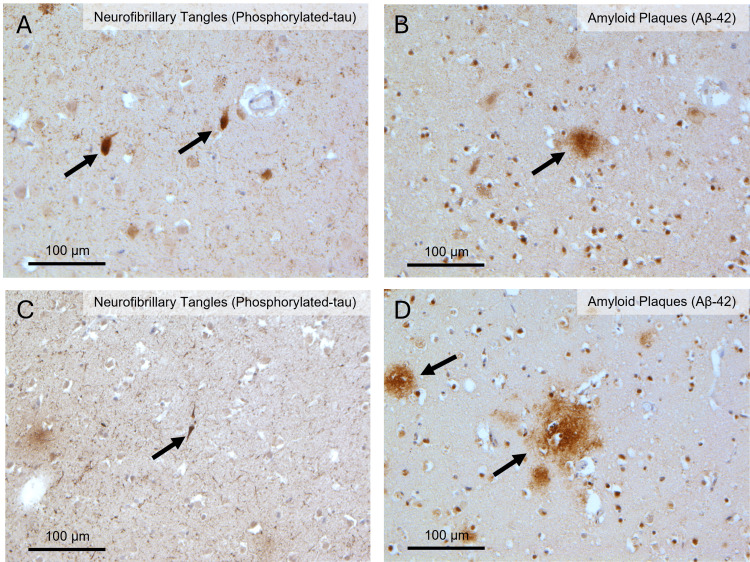
Alzheimer’s disease-related pathology in the hippocampus and cerebral hemisphere of donor 2 (D2). (A) Hippocampal section stained for phosphorylated tau showing intraneuronal neurofibrillary tangles with characteristic elongated and fibrillar morphology (black arrows). (B) Cerebral hemisphere section stained for Aβ-42 demonstrating a dense-core extracellular amyloid plaque (black arrow). (C) Cerebral hemisphere section stained for phosphorylated tau showing a fibrillar neurofibrillary tangle within the cortical neuropil (black arrow). (D) Cerebral hemisphere section stained for Aβ-42 demonstrating multiple extracellular amyloid plaques, including a large dense-core plaque and a smaller plaque deposit (black arrows). Aβ-42: amyloid beta 42

**Figure 3:**
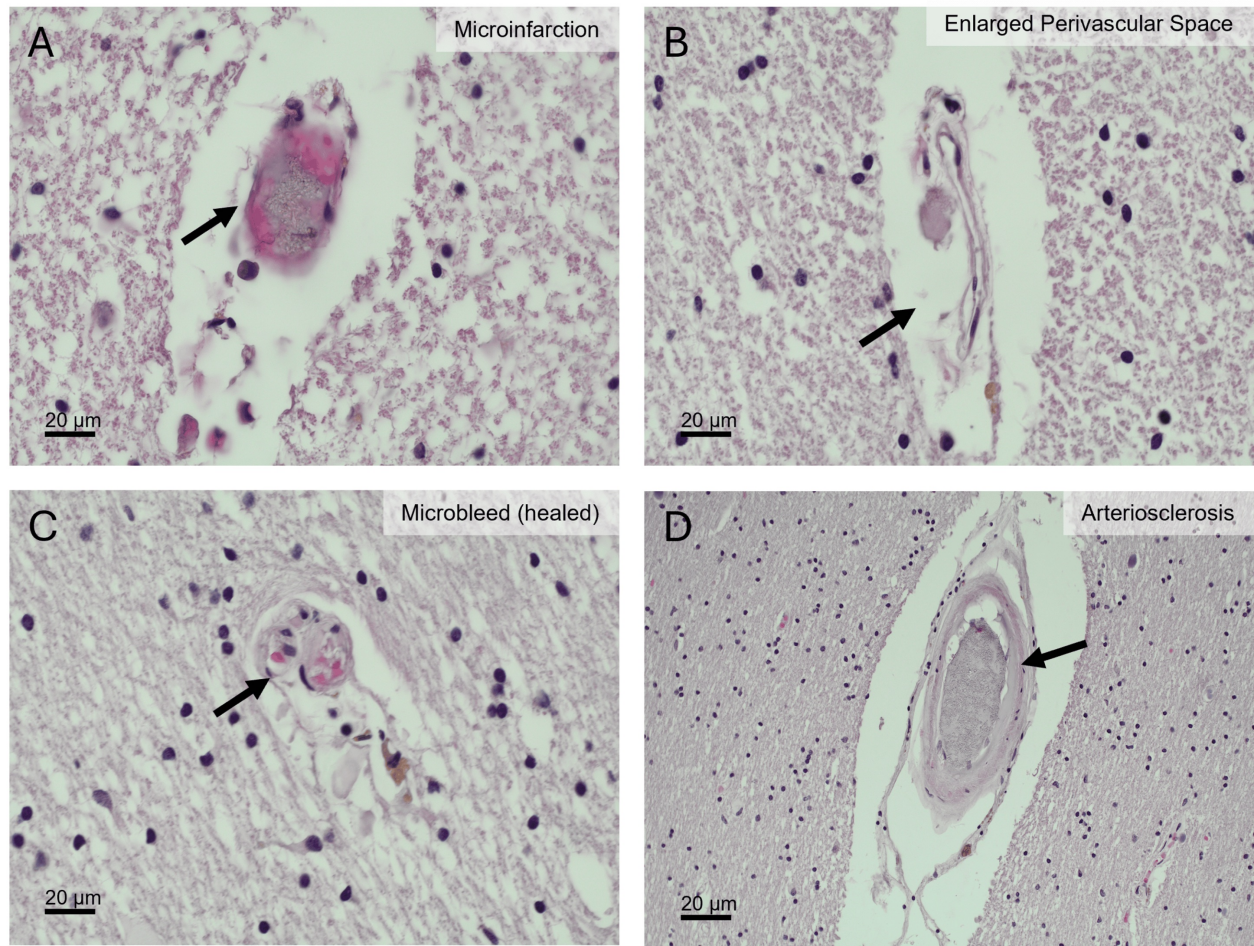
Cerebrovascular pathology in the cerebral hemisphere of donor 1 (D1). (A) Section showing a focal microinfarct characterized by localized tissue necrosis and eosinophilic debris adjacent to a small vessel (black arrow). (B) Section demonstrating an enlarged perivascular space surrounding a small penetrating vessel (black arrow), indicative of impaired perivascular drainage and small vessel disease. (C) Section showing a healed microbleed with residual hemosiderin deposition within the vessel wall and surrounding parenchyma (black arrow). (D) Section demonstrating arteriosclerotic vessel wall thickening with concentric narrowing of the vascular lumen (black arrow), consistent with chronic small vessel arteriosclerosis.

**Figure 4:**
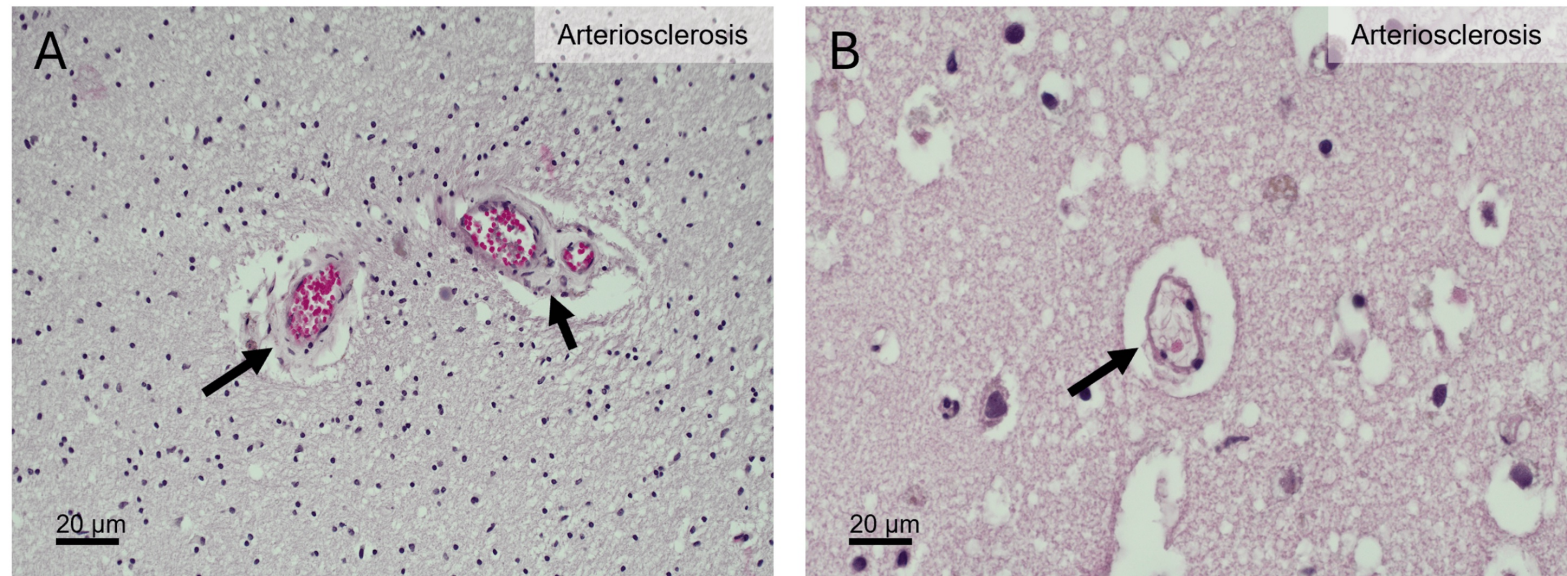
Cerebrovascular pathology in the cerebral hemisphere of donor 2 (D2). (A) Section showing the arteriosclerotic thickening of small vessel walls with concentric narrowing of the vascular lumen (black arrows), indicating moderate small vessel arteriosclerosis. (B) Section demonstrating a sclerotic small artery with marked wall thickening and luminal narrowing (black arrow), consistent with advanced arteriosclerotic remodeling.

A thorough histochemical (hematoxylin and eosin, H&E) and immunohistochemical (β-amyloid and tau protein) examination of D1 and D2 brains performed as previously described revealed the presence of AD-related pathology in both individuals, with AD stages being mild in D1 (Figure 1) and intermediate in D2 (Figure 2) [15]. The cVD-related pathology was also evident in both cases and was characterized by several microbleeds indicative of a compromised BBB integrity in D1 (Figure 3). Blood vessel wall thickening characteristic of arteriosclerosis was significant in D1 (Figure 3) but less so in D2 (Figure 4). These data, alongside the RA diagnoses, are consistent with the coexistence in both donors of three major diseases: RA, cVD, and AD.

Genetic screening

The probing of the DNA protein-coding regions (exons) by whole exome sequencing (WES) on the next-generation sequencing (NGS) Illumina platform (San Diego, CA) and the respective bioinformatics analysis were performed as previously described [16]. The cumulative exome coverage for the >25× depth of coverage for D1 and D2 was, respectively, 92% and 97%. Functional annotation of genes with rare (minor allele frequency {MAF} ≤ 0.01) pathological/deleterious variants was performed by searching GeneCards, Google Scholar, and PubMed databases.

The genetic screening by WES yielded 94 (D1) and 99 (D2) mutated genes with rare pathological/deleterious variants that were grouped into 17 categories most relevant to the present case, including those linked to arthritis, cerebral vasculature, and dementia pathologies (Tables 1, 2). Such gene grouping was consistent with the donors’ diagnoses, as well as the results of neuropathological examination. The genetic screening also revealed seven mutated genes with rare pathological/deleterious variants that were shared by both donors. Importantly, six of those genes had identical variant matches in D1 and D2 (Table 3).

**Table 1:** Genes with rare pathological/deleterious variants in D1 grouped into the categories most relevant to the present case. D1: donor 1

Categories	Genes
Rheumatoid Arthritis	*CD274, CEL, CEP131, COL22A1, CPOX, DENND1C, DNAH8, DNAJB6, FAM83A, FAT3, HLA-DRB1, HOXB2, HYDIN, IGSF3, KIAA1549L, KIR2DL4, MC1R, MTUS1, MUC16, NDUFV1, NEK1, NOP10, NUMA1, OBSL1, OMA1, PANK4, PCDHA4, PCK2, PDGFRL, PGM5, PLB1, PRSS1, SLIT1, SNTA1, SYNE1, *and* USP44*
Juvenile Idiopathic Arthritis	*COL28A1, OBSL1, PGM5, RNF135, SERPINB2, USH2A, *and* WASF2*
Psoriatic Arthritis/Psoriasis	SYNE1
Osteoarthritis	*AQP7, COL22A1, CPOX, HLA-DRB1, HLA-DRB5, KCNJ12, KIAA1549L, MTUS1, MYH1, NDUFV1, PGM5, *and* SPATC1*
Lupus	*ARSD, NUMA1, *and* OBSL1*
Sjögren’s Syndrome	*HLA-DRB5, KCNH6, NUMA1, *and* PCDHA4*
Ankylosing Spondylitis	*GPR25 *and* NEK1*
Bone Physiology	*ACSM4, ALS2CL, ANKRD12, ARSD, CAPRIN2, KIF1C, SERPINB2, SGIP1, SLC9A6, *and* WASF2*
Cartilage Physiology	*ARSD, COL22A1, FAT3, HYDIN, NMBR, PCDHA4, *and* PGM5*
Cerebral Vasculature/Stroke	*AQP7, FAM160A1, GPR25, HOXB2, IGSF3, KCNJ12, MAML2, NOP10, NUMA1, SERPINB2, SGIP1, *and* SNTA1 *
Senescence/Aging	*ARHGEF5, CLASP2, *and* MTUS1*
Cognitive Impairment/Dementia	*ACOT8, ACSM4, CD274, CEP131, CEP131, CEP135, COL28A1, DNAH8, ELP2, HLA-DRB1, HLA-DRB5, MAML2, NCAPG2, NDUFV1, NMBR, OBSL1, OTOF, OTOP1, PANK4, PCDHA4, SLC9A6, *and* SYNE1*
Alzheimer’s Disease	*ACOT8, ANKRD12, AQP7, CAPRIN2, CD274, COL22A1, COL28A1, CPOX, DNAH8, FAM160A1, HLA-DRB1, HLA-DRB5, HOXB2, HYDIN, IGSF3, KCNH6, MAML2, MC1R, MTUS1, MUC16, NUMA1, OTOF, PCDHA4, PCDHB11, PCDHB8, PCK2, PDGFRL, PRSS1, SGIP1, SLIT1, SNTA1,UHRF1BP1L, *and* WDR86 *
Parkinson’s Disease	*FAM160A1, FAM83A, MYH1, NCAPG2, NDUFV1, NUMA1, OMA1, PAH, *and* UHRF1BP1L *
Vascular Dementia	*ARMC6, CAPRIN2, NUMA1, PRSS1, *and* SLIT1*
Neurodevelopment	*BRAT1, CAPRIN2, ELP2, FAT3, *and* SLIT1*
Atherosclerosis	*BRAT1, CAPRIN2, *and* CLASP2*

**Table 2:** Genes with rare pathological/deleterious variants in D2 grouped into the categories most relevant to the present case. D2: donor 2

Categories	Genes
Rheumatoid Arthritis	*ALDH9A1, BHMT, C8A, CEP350, CHST4, COL15A1, DPEP3, FAM81A, FOXRED1, GBE1, GGT1, GHRL, GRIN3B, HYDIN, IGSF3, IL10RA, KCNE2, LAMC3, LAPTM5, MXRA5, PER3, PIK3IP1, PRR14L, PRSS1, PTPN7, PXDNL, SRCAP, TRPV1, *and* UNC45B*
Juvenile Idiopathic Arthritis	*GP1BA, IL10RA, NOC4L, SRCAP, TRHR, *and* ZIK1*
Psoriatic Arthritis/Psoriasis	*SIDT1 *and* SRRM1*
Osteoarthritis	*ALDH9A1, AQP7, B3GNT9, CEP350, COL15A1, CRAT, FAM81A, GBE1, GP1BA, LAMC3, MXRA5, PER3, PRR14L, PTPN7, *and* PXDNL*
Lupus	*ARSD, GGT1, *and* ZMYND8*
Sjögren’s Syndrome	*C9orf131 *and* CEP350*
Ankylosing Spondylitis	*DPEP3, NOC4L, *and* SRRM1*
Bone Physiology	*DAAM2, FAM160A1, FAM160A1, GGT1, LRP5L, LSG1, *and* PROSER2*
Cartilage Physiology	*ARSD, B3GNT9, CRAT, CYB5D1, KRTAP4-11, *and* MXRA5*
Cerebral Vasculature/Stroke	*AQP7, CEP350, EBNA1BP2, FAM160A1, FAM160A1, FOXS1, GP1BA, IL10RA, *and* LSG1*
Senescence/Aging	KCNE2
Cognitive Impairment/Dementia	*CC2D1A, CEP350, COL15A1, DNAH10, EIF2B5, GBE1, GGT1, GHRL, KCNE2, LAPTM5, OTOP1, UNC45B, ZMYND8, *and* ZNF256*
Alzheimer’s Disease	*ALDH9A1, AQP7, BHMT, CRB2, CRB3, DAAM2, DNAH10, EBNA1BP2, FAM81A, FRMD1, GGT1, GHRL, GP1BA, HYDIN, IGSF3, INSRR, LAMC3, LAPTM5, LRP5L, MEP1A, MRPL23, NOC4L, NTSR1, PCDHGA8, PIK3IP1, PRKD2, PRSS1, RTN4RL1, SHARPIN, SIGLEC11, SRCAP, SRRM1, TBC1D26, TRHR, TRIOBP, WDR7, ZMYND8, *and* ZNF256*
Parkinson’s Disease	*ALDH9A1, FAT2, GBE1, MMRN1, MRPL23, PXDNL, RAB25, SRRM1, TEKT4, *and* TRPV1*
Vascular Dementia	*ALDH9A1, B3GNT9, BHMT, DAAM2, GGT1, PRSS1, PTPN7, TOP3A, *and* TRPV1*
Neurodevelopment	*CC2D1A, CRAT, DAAM2, EIF5AL1, FAAH2, FAT2, GBE1, GRIN3B, LAMC3, PCDHGA8, RTN4RL1, SRCAP, TRIOBP, *and* ZNF430*
Atherosclerosis	LSG1

**Table 3:** Shared genes mutated in D1 and D2 and their respective rare pathological/deleterious variants*. *Gene-to-protein name conversion was performed by using the GeneCards database. MAF, minor allele frequency; D1, donor 1; D2, donor 2

Genes	Proteins	Variants	MAF
AQP7	Aquaporin-7	D1/D2:NM_001318156:exon4:c.T172C:p.Y58H;NM_001318158:exon5:c.T343C:p.Y115H	0.0073
ARSD	Arylsulfatase D	D1/D2:NM_001669:exon6:c.G959A:p.G320D	0.0001
FAM160A1	FAM160A1	D1:NM_001348694:exon13:c.C2981G:p.P994R	0.098
		D2:NM_001348694:exon3:c.C11T:p.S4L	1.28E-05
HYDIN	Hydrocephalus-Inducing Protein Homolog	D1/D2:NM_001270974:exon40:c.C6259T: p.R2087C	1.61E-05
IGSF3	Immunoglobulin Superfamily Member 3	NM_001007237:exon7:c.C1951T:p.R651W and NM_001542:exon8:c.C2011T:p.R671W	0.0026
OTOP1	Proton Channel OTOP1	NM_177998:exon6:c.G1793C:p.R598P	0.0073
PRSS1	Trypsin-1	D1/D2:NM_002769:exon2:c.G146T:p.G49V	0.0079
		D1/D2:NM_002769:exon2:c.T158A:p.I53N	0.0005
		D1/D2:NM_002769:exon2:c.C162G:p.N54K	0.0002
		D2:NM_002769:exon4:c.A547G:p.M183V	4.27E-06

## Discussion

The results of the neuropathological examination and the donors’ medical history point toward the coexistence of three major diseases: RA, cVD, and AD in the present case. The genetic screening provided two important insights pertinent to the molecular underpinnings of such coexistence in humans. First, the presence of multiple respective mutated genes linked to each of the above diseases was consistent with their polygenic nature and could point to the possibility of their independent occurrence. Second, the genetic screening also identified seven affected genes with rare pathological/deleterious mutations, *AQP7*, *ARSD*, *FAM160A1*, *HYDIN*, *IGSF3*, *OTOP1*, and *PRSS1*, that were shared by both donors with identical variant matches except for *FAM160A1*. Interestingly, three of those shared genes, *FAM160A1*, *IGSF3*, and *PRSS1*, appeared to be pleiotropic as they could be linked to either RA [17-21], cVD [22-24], or AD [25-28]. Therefore, a contribution of pleiotropic genetic component to the coexistence of the above pathologies and, therefore, the presence of their common risk factor(s) should also be considered.

Another important piece of information obtained from the genetic screening was related to RA in the donors. Unexpectedly, in addition to mutated genes linked to RA, multiple affected genes associated with osteoarthritis and juvenile idiopathic arthritis, as well as the genes known to be involved in the regulation of bone and cartilage physiology, were also found in D1 and D2. The mutations of the latter could target joints directly, independently of RA, and may be one of the reasons for hip replacement surgery performed bilaterally in D1 and unilaterally in D2. Intriguingly, besides the genes associated with RA in both donors, there were also mutated genes linked to systemic lupus erythematosus (SLE), Sjögren’s syndrome (SS), and ankylosing spondylitis (AS). The coexistence of RA, SLE, SS, and AS has been recently reported, which raises a question regarding the potential contribution of undiagnosed concurrent non-RA autoimmune and inflammatory diseases to the RA clinical presentation [29]. Yet, the coexisting autoimmune diseases such as RA and SLE could mutually promote AD development due to their strong association with AD [30].

## Conclusions

The data presented herein may indicate that the coexistence of AD, cVD, and RA in humans could be underpinned by a combination of polygenic and pleiotropic factors. The RA clinical presentation could also be influenced by concurrent autoimmune and inflammatory diseases, and the severity of the joint damage in RA may also be dependent on genetic mutations directly targeting the bones and cartilage. Because of the limited number of individuals involved in the present case, the respective results and conclusions should be viewed as an essential starting point for follow-up clinical studies with a large patient cohort.
